# The Role of Item-Specific Information for the Retrieval Awareness of Performed Actions

**DOI:** 10.3389/fpsyg.2018.01325

**Published:** 2018-08-14

**Authors:** Guangzheng Li, Lijuan Wang

**Affiliations:** ^1^School of Education Science, Jiangsu Normal University, Xuzhou, China; ^2^School of Psychology, Northeast Normal University, Changchun, China

**Keywords:** subject-performed task, verbal task, *remember/know* judgments, retrieval awareness, item-specific information

## Abstract

Research on action memory has been pursued for more than 30 years, but it is still unclear what drives the recollection process of performed actions. In this study, we used the *remember/know* paradigm and designed two experiments to examine the relation between item-specific processing and retrieval awareness of subject-performed tasks (SPT). The results showed that SPT allows *remember* responses in *remember-know* judgments more easily; that is, SPT can enhance the frequency of recalling re-collective experience. Item-specific processing can improve the memory performance and the proportion of *remember* judgments of verbal tasks (VT), but it does not improve the memory performance and proportion of *remember* judgments of SPT, indicating that SPT can enhance item-specific processing, which leads to more *remember* responses in judgment. The relation between item-specific processing and retrieval awareness of SPT is also discussed.

## Introduction

Memory for simple action phrases (such as “pick up the pencil” or “break the stick”) are retained better when the participants are instructed to learn the phrases while enacting them rather than just listening or reading. The excellent memory performance in subject-performed tasks (SPT), as compared to verbal tasks (VT), has been called the “enactment effect” or “SPT effect” (for reviews see Cohen, 1989; Engelkamp, 1998; Zimmer et al., 2007). This effect is reliable and robust and has been observed in a variety of encoding and test conditions. It is observed whether the subjects are performing actions with actual objects or only using gestures (for reviews see Madan and Singhal, 2012a), whether a recall or recognition test is used (Cohen, 1989), and whether the subjects are young children or old people (Feyereisen, 2009; Mecklenbräuker et al., 2011). In a large sample (*N* = 1,000) analysis, about 88% of subjects showed significant SPT effects, and the degree of memory enhancement following encoding enactment is substantial, often in the range of 20–30% (Nyberg et al., 2002). Although the SPT effect has been studied for more than 30 years, it has not been determined what drives the recollection process for performed actions. In the present study, we investigate this issue.

### Retrieval awareness of performed actions

Episodic memory is the ability to consciously remember one's experiences of events that occurred at a particular time and place and is typically characterized by temporal and spatial details (Tulving, 1985). Tulving (2002, 1985), associated different levels of memory systems with different levels of consciousness and connected episodic memory with the highest level of human consciousness—auto-noetic consciousness. Tulving (2002) pointed out that the individual who is engaged in the subjective re-experience of the event, accompanied by a personal emotional state, is undergoing auto-noetic consciousness. Semantic memory, conversely, involves the general concepts, and knowledge of the world that are shared by others; thus, the memory is not accompanied by personal feelings but only by noetic consciousness.

Previous studies have often adopted the *remember/know* paradigm to test auto-noetic consciousness and noetic consciousness (Tulving, 1985; Gardiner, 1988, 2001). The procedure involves a recognition test, where people are asked to identify the basis on which they judged that an item had been previously studied—whether they “remembered” its prior occurrence or simply “knew” on some other basis that it was old (Tulving, 1985; Gardiner, 2001). A *remember* response is taken to indicate a state of auto-noetic awareness, defined as the subjects being consciously aware of some aspects of what they experienced when the item was first learned (Tulving, 1985; for a more detailed discussion see Gardiner, 2001). A *know* response is taken to indicate a state of noetic awareness, defined as recognition that the item had been presented earlier but without the ability to recollect consciously anything about its actual occurrence or what the subject experienced at that time (Tulving, 1985; for a more detailed discussion see Gardiner, 2001).

This aspect is often overlooked while researching into the SPT effect. Although Kormi-Nouri (1995) proposed the episodic integration theory to explain it and suggested that a good self-involvement level accompanied by enactment caused the effect, this study did not provide evidence. To our knowledge, only a handful of studies have examined the retrieval awareness in SPT. The first study of the re-collective experience of SPT was reported by Conway and Dewhurst (1995). In their experiment, the subjects studied a series of phrases by performing, watching, or imagining them. Then in a recognition test, they were asked to judge whether their recognitions were made on the basis of conscious re-collective experience (*remember* responses), or on some other basis such as familiarity (*know* responses). The authors found that the proportion of correct *remember* responses was much higher for performed actions than for observed actions (see also Manzi and Nigro, 2008). However, because both studies presented corresponding objects during the performing actions, it is not possible to determine whether the *remember* responses occurred more frequently for performed actions because of the enactment or because of the abundant sensory information brought by the objects. Engelkamp and Dehn (1997) solved this problem and compared the relative contributions of familiarity and recollection to recognition following SPT and VT without the presence of the relevant objects. They found an increase in *remember* judgments after SPT as compared to VT and concluded that the SPT effect is due mainly to an enhanced recollection process after SPT (see also Lövdén et al., 2002).

In spite of this, it is still not clear what drives the recollection process of SPT. Some researchers pointed out that the characteristics of the encoding shape the recollection process of SPT (Zimmer et al., 2001; Magnussen and Helstrup, 2007), but these studies were all reviews and did not provide empirical support. Gardiner et al. (2006) found that the recognition performance and the *remember* responses were not enhanced by SPT in a case of developmental amnesia in which the episodic memory was impaired. More recently, Zhao et al. (2016) pointed out that enactment unites the components of actions so that familiarity can support associative recognition following SPT; however, their study did not provide behavioral evidence. Therefore, the purpose of the present study is to use the *remember/know* paradigm and make VT a control condition to explore what drives the recollection process of SPT.

### Item-specific processing of performed actions

Item-specific information involves the characteristics of an item that makes it unique and discriminable from other items that are studied together with it. Most of the research suggests that SPT promotes item-specific knowledge, making the item more specific and distinguishable (e.g., Steffens et al., 2006, 2009; Kubik et al., 2016). The item-specific information accompanied by SPT can be especially effective in leading to the memory advantage of encoding by enacting (Seiler and Engelkamp, 2003; Schatz et al., 2011). The assumption of enhanced item-specific processing is based on the result that the enactment effect is much more notable in recognition and cued-recall than in free-recall (Steffens et al., 2006, 2009). These findings have been explained by the fact that different ways of testing depend on different processing mechanisms. Recognition and cued-recall tests mainly examine, respectively, the feeling of familiarity with the item and the degree of connection between the clue and the target word, which reflects item-specific information. However, free-recall tests also require relational information, which is generally not heightened by SPT over VT when no action-related objects are presented (Engelkamp et al., 2005).

For example, using lists of independent items, Steffens et al. (2009) discovered that the parameter measuring the item-specific processing was enhanced. They argued that SPT is more complete in the semantic-related properties of action phrases. Engelkamp (1995) pointed out that the participants focus their attention on the enactment task and ignore irrelevant environmental details while the action is being performed. As a result, they gain more details. Kormi-Nouri (1995) suggested that during the execution of the action, participants focus on the verb, the noun, and the whole action phrase, so that the information in the action phrase is fully processed by enactment. Moreover, on this basis, Steffens et al. (2006) argued that enactment can enhance item-specific information mainly in the following three respects: (1) the item-specific information of the noun, (2) the item-specific information of the verb, and (3) the integration of the verb and the noun (which they called “retrieval”; see Steffens et al., 2006, 2009, for more details). These results have been supported by subsequent studies (Kubik et al., 2014a,b; Schult et al., 2014; Mulligan and Peterson, 2015).

As mentioned above, most of the studies of item-specific processing focused on whether SPT enhances item-specific information, and no studies have explored the relationship between the item-specific processing and retrieval awareness. For this reason, the current study did not present the corresponding objects in the experiments, and used VT as the comparison group, to explore the retrieval awareness of SPT. Experiment 1 used the *remember/know* paradigm to verify that SPT is accompanied by more re-collective retrieval. Experiment 2 examined the hypothesis that item-specific processing leads to more *remember* responses.

## Experiment 1

The aim of Experiment 1 was to use the *remember/know* paradigm to verify the existing research conclusions. We assumed that a reliable SPT effect would be obtained and more *remember* responses would occur for SPT than for VT.

### Methods

#### Subjects and experimental design

Prior to the experiment, we used G^*^Power (Erdfelder et al., 1996) to calculate the sample size, which was sufficient to obtain 0.80 power for a 0.05 probability error. In this experiment, we expected an effect size of 0.45 and a correlation among repeated measures of 0.5. The sample size was 19 subjects. We recruited 20 students (7 males and 13 females, *M*_age_ = 22.50, *SD* = 1.20) as participants from Jilin University in China. A single within-subjects design was used for the study. The protocol was approved by the Ethnic Committee of Jilin University, and written consent was obtained from all the participants before conducting the test.

#### Materials

We selected 96 action phrases (e.g., “open the textbook,” and “pick up the pencil”, see Table A1) that were roughly matched in word length (2–4 Chinese characters). Half the phrases (48) were used for study, and the other half (48) were used for counter-balanced distractors. The phrases used in the study were independent of parts of the body (e.g., “scratching the head”) and did not refer to objects in the laboratory (e.g., “banging on the table”), because these types of phrases would have clear retrieval clues and could be recalled easily, thereby interfering with the SPT effect (Steffens et al., 2007).

We randomly recruited 17 additional students to assess the familiarity of the phrases before the experiment on a 7-point scale (1 indicating lowest familiarity, 7 indicating highest familiarity). One-way repeated measures analysis of variance (ANOVA) was used to assess the results; the difference in familiarity between the learning materials (*M* = 4.26, *SD* = 0.85) and the interfering materials (*M* = 4.17, *SD* = 0.79) was not significant, *F*_(1, 16)_ = 1.26, *MSE* = 0.057, *p* = 0.286, and η^2^_*p*_ = 0.07.

#### Procedure

The experimental procedure had two stages, learning and testing, which were compiled by E-prime 2.0. Each study trial began with a fixation cross (+) displayed at the center of the screen for 2,000 ms. An action phrase was then centrally displayed against a white background for 6,000 ms. The subjects were told to study the items for the subsequent recognition test. Half the learning words (24) were studied in SPT: the subjects were asked to read the phrases silently first and then pretend to perform the actions with imaginary objects. The other half (24) were studied in VT: the subjects were asked to read the phrases silently but were prohibited from making unnecessary hand movements. For the learning sequence, half of the subjects first studied in SPT and then in VT, while the other half took the reverse order. After the last word was presented, the subjects were told to solve four funny math problems in 5 min, and then the recognition test was conducted.

In the test phase, old and new items were randomly presented to the subjects on the computer screen. The subjects were asked to determine whether the items were new (key labeled X) or old (key labeled J). If they were sure that the item was old, they were required to make a *remember/know* judgment by pressing the “R” or “K” key within 6 s. They were told to press “R” if they consciously remembered the items and could recall the details of learning it (such as the number of the silent reading, the magnitude of the action, the learning mood, the items before or after it, and if the phrase let the subject re-experience the past). They were told to press “K” if they were sure that the word had been presented but could not recollect its actual occurrence or any related details. If they were sure that the word was new, the *remember/know* judgment was not applicable, and they were required to press the “X” key. All the subjects were required to keep their fingers on the keys throughout the experiment and to respond to the tasks as quickly as possible without sacrificing accuracy. The entire experiment lasted for about 15–20 min.

#### Data calculation method

This study adopted the *remember/know* recognition paradigm and used the method of signal detection to calculate hit rates and false-alarm rates. In all the following analyses, a hit was defined as responding “old” to a repeated word, and a false alarm as responding “old” to a new word. We could not calculate the false-alarm rates under every specific encoding condition because of the within-subjects design. Therefore, we only calculated the false-alarm rates of the recognition test and the *remember/know* judgment.

### Results

Table 1 presents the mean proportions of hits and false alarms under different study conditions and response types.

**Table 1:** Mean proportion of hits and false alarms as a function of study conditions and response type in Experiment 1.

	**SPT**		**VT**		**False alarms**	
	***M***	***SD***	***M***	***SD***	***M***	***SD***
Recognition	0.93	0.07	0.79	0.17	0.05	0.04
R	0.86	0.22	0.63	0.29	0.02	0.02
K	0.07	0.21	0.15	0.20	0.03	0.04

A one-way ANOVA was done to explore the order effect (SPT – VT vs. VT – SPT) on the recognition performance of SPT and VT, respectively. The order effect did not have a significant difference on SPT and VT. The corresponding values were, respectively, *F*_(1, 18)_ = 2.31, *p* = 0.15, η^2^_*p*_ = 0.11, and *F*_(1, 18)_ = 0.98, *p* = 0.36, η^2^_*p*_ = 0.05. For completeness, we also adopted a non-parametric statistical approach and found similar results.

One-way repeated measures ANOVA was used for recognition performance. The result showed that the main effect of the encoding condition was significant, *F*_(1, 19)_ = 20.37, *MSE* = 0.009, *p* = 0.001, and η^2^_*p*_ = 0.52, thereby indicating that the performance of SPT was significantly better than that of VT.

One-way repeated measures ANOVA was used for *remember* responses. The results showed a significant main effect of the encoding condition, *F*_(1, 19)_ = 17.12, *MSE* = 0.029, *p* < 0.01, and η^2^_*p*_ = 0.47, thereby demonstrating that more *remember* responses were given to the items in SPT than in VT. However, the effect was reversed for *know* responses: more “K” responses were given to the items in VT than in SPT, *F*_(1, 19)_ = 4.95, *MSE* = 0.017, *p* < 0.05, and η^2^_*p*_ = 0.21.

### Discussion

For the recognition performance, the study demonstrated a significant SPT effect. This supports the previous results that the SPT effect is stable, and this stability does not depend on the test method or on the experimental design type (Roediger and Zaromb, 2010). For the *remember/know* judgment, more *remember* responses were given to the items in SPT than in VT. This is also similar to the previous results. Earlier studies have shown that SPT engenders *remember* responses more easily than Experimenter Performed Tasks (EPT) (Conway and Dewhurst, 1995; Manzi and Nigro, 2008) and VT (Engelkamp and Dehn, 1997), and that different encoding types can stably influence the frequency of recalling re-collective experiences. In this study, the difference between SPT and VT was that subjects in SPT were required to perform corresponding actions while those in VT were not. Therefore, we suggest that performing an action constitutes an episodic event, which allows participants to retrieve more specific information. If the specific information is more abundant in the encoding, there will be more retrieval cues.

## Experiment 2

This experiment was conducted to verify the results of Experiment 1. However, we adopted the *remember/know* paradigm to evaluate the hypothesis that item-specific processing leads to more *remember* responses. Specifically, the experiment was based on the following two considerations: First, because of the within-subjects design of Experiment 1, we could not calculate the false-alarm rates under each encoding condition. To overcome this problem, in Experiment 2 we used a between-subjects design, and this also allowed us to verify whether the experimental design affects the results. Second, the *remember/know* judgment was based directly on whether the subject had specific information about the learned item, and this paradigm was more straightforward for measuring item-specific information. To enhance the item-specific processing, the subjects were asked to rate the pleasantness of each action phrase; this technique is frequently used in orienting tasks to focus the subject on item-specific information (e.g., Seiler and Engelkamp, 2003).

*Remember* responses are often associated with item-specific information. Therefore, it can be speculated that if the participants pay attention to item-specific information during the encoding, they will make more *remember* responses in the judgment. If SPT enhances item-specific processing, and the participants recall more details of the items, *and* their memory performance is better, then the additional item-specific processing cannot enhance the memory performance as well as the *remember* responses of SPT. On the other hand, the item-specific information is assumed to play a smaller role in VT, so the pleasantness rating should enhance the item-specific processing. Encoding and retrieval processes in VT should approximate those of SPT, and the memory performance as well as the *remember* responses should be improved in VT.

### Methods

#### Experimental design

A 2 (encoding conditions: SPT and VT) × 2 (instructional methods: standard instruction and pleasantness rating) between-subjects design was used.

#### Subjects and materials

Because of the between-subjects design, we expected an effect size of 0.3. The sample size required was 80 subjects, with 20 in each condition. Therefore, we recruited 80 participants (47 males and 33 females, *M*_age_ = 22.08, *SD* = 1.20) from Jilin University in China. The materials were same as those in Experiment 1.

#### Procedure

The procedure was basically the same as that in Experiment 1. However, we added the pleasantness rating task in addition to the standard instruction condition: the subjects were asked to evaluate the pleasantness of each action phrase on a five-point scale (ranging from 1 = unpleasant to 5 = pleasant) during the study, both in VT and SPT. They were required to make a snap decision. In SPT, the rating followed the enactment.

#### Data calculation method

The data analysis was also similar to that of Experiment 1, except that we adopted a between-subjects design. This let us calculate the rates of hits and false alarms under different conditions. The discriminability index (d′ = Z _hit rates_– Z _false alarm rates_) was then calculated as the dependent variable measuring performance on recognition and *remember* responses. The larger the d' value in the discriminability index, the higher the sensitivity and the better the performance of the subjects; the lower the d' value, the lower the sensitivity and the poorer the performance.

### Results

Prior to dealing with the data, we first analyzed whether there was a significant difference in the pleasantness ratings between SPT and VT. A One-way ANOVA showed that there was no significant difference, *F*_(1, 38)_ = 1.04, *MSE* = 0.20, *p* = 0.31, and $\eta_{\text{p}}^{2}$ = 0.03

#### Recognition results

A 2 × 2 (encoding method × instruction) between-subjects ANOVA was conducted to investigate the pattern of the discriminability index shown in Table 2. The main effect of the encoding method was significant, *F*_(1, 76)_ = 8.76, *MSE* = 0.56, *p* = 0.004, and $\eta_{\text{p}}^{2}$ = 0.10, with better performance for SPT than for VT. The main effect of instruction was not significant, *F*_(1, 76)_ = 2.23, *MSE* = 0.56, *p* = 0.13, and $\eta_{\text{p}}^{2}$ = 0.03. The interaction between the encoding and instruction conditions was significant, *F*_(1, 76)_ = 9.33, *MSE* = 0.56, *p* = 0.003, and $\eta_{\text{p}}^{2}$ = 0.11. Simple main-effect tests revealed that memory performance was significantly better in the pleasantness-rating instruction condition than in the standard condition for the VT group, *F*_(1, 76)_ = 10.33, *MSE* = 0.56, *p* = 0.002, and $\eta_{\text{p}}^{2}$ = 0.12. However, no such difference emerged in the SPT groups, *F*_(1, 76)_ = 1.22, *MSE* = 0.56, *p* = 0.27, and $\eta_{\text{p}}^{2}$ = 0.02. The above results indicate that the pleasantness-rating instruction improves the performance only in VT.

**Table 2:** Table shows the mean proportion of hit rates and false-alarm rates, the discriminability index (d′ = Z _hit rates_– Z _false−alarm rates_), and the standard deviation in each study condition and response type.

	**Standard condition**				**Pleasantness rating condition**			
	**SPT**		**VT**		**SPT**		**VT**	
	***M***	***SD***	***M***	***SD***	***M***	***SD***	***M***	***SD***
Hits	0.94	0.06	0.83	0.13	0.93	0.05	0.92	0.09
False alarms	0.03	0.03	0.09	0.12	0.05	0.05	0.06	0.08
d′	3.65	0.56	2.65	0.73	3.40	0.61	3.41	1.00

#### Results for remember/know judgment

A 2 × 2 (encoding × instruction) between-subjects ANOVA was conducted to explore the pattern of the discriminability index of the *remember* response, as shown in Table 3. There was a significant main effect of the encoding method, *F*_(1, 76)_ = 8.18, *MSE* = 0.74, *p* < 0.005, and $\eta_{\text{p}}^{2}$ = 0.10. The main effect of instruction was not significant, *F*_(1, 76)_ = 1.36, *MSE* = 0.74, *p* = 0.25, and $\eta_{\text{p}}^{2}$ = 0.02. The interaction between encoding and instruction conditions was significant, *F*_(1, 76)_ = 11.61, *MSE* = 0.74, *p* = 0.01, and $\eta_{\text{p}}^{2}$ = 0.13.

**Table 3:** Table shows the mean proportions of hit rates and false-alarm rates, the discriminability index (d′ = Z _hit rates_– Z _false−alarm rates_), and the standard deviation in each study condition and response type.

	**Standard condition**				**Pleasantness rating condition**			
	**SPT**		**VT**		**SPT**		**VT**	
	***M***	***SD***	***M***	***SD***	***M***	***SD***	***M***	***SD***
**REMEMBER**								
Hits	0.86	0.21	0.64	0.28	0.85	0.12	0.84	0.13
False alarms	0.01	0.02	0.05	0.07	0.04	0.05	0.03	0.05
d′	3.44	0.98	2.23	0.98	3.01	0.16	3.11	0.91
**KNOW**								
Hits	0.07	0.22	0.2	0.21	0.08	0.1	0.07	0.08
False alarms	0.007	0.01	0.04	0.05	0.01	0.02	0.01	0.03
d′	0.07	0.22	0.17	0.21	0.07	0.10	0.06	0.07

Simple main-effect tests revealed that the participants made more *remember* responses under the pleasantness-rating instruction than under standard instruction in the VT group, *F*_(1, 38)_ = 10.48.1, *MSE* = 0.74, *p* = 0.02, and η^2^_*p*_ = 0.12, but not in the SPT group, *F*_(1, 38)_ = 2.50, *MSE* = 0.74, *p* = 0.12, and η^2^_*p*_ = 0.03. This shows that the pleasantness-rating instruction significantly increased the incidence of *remember* responses for VT group, but not for SPT group.

However, for the K response, the main effects of encoding method and instruction were not significant. The corresponding values were *F*_(1, 76)_ = 0.78, *MSE* = 0.62, *p* = 0.38, and η^2^_*p*_ = 0.01; and *F*_(1, 76)_ = 0.25, *MSE* = 0.62, *p* = 0.62, and η^2^_*p*_ = 0.03. The interaction between the encoding method and instruction was not significant, *F*_(1, 76)_ = 1.46, *MSE* = 0.62, *p* = 0.23, and η^2^_*p*_ = 0.02.

### Discussion

The aim of Experiment 2 was to verify the results of Experiment 1 and examine the hypothesis that item-specific processing leads to more *remember* responses in SPT. The results confirmed this expectation. On the one hand, SPT produced better recognition performance and engendered more *remember* responses under standard instruction, independent of design type. The recognition performance was consistent with previous results showing that the SPT effect is reliable. It was observed whether a between-subjects or within-subjects design was used, and whether the control group was VT or EPT (Engelkamp and Dehn, 2000). On the other hand, the pleasantness-rating instruction significantly increased the incidence of *remember* responses and the recognition performance for the VT group but not the SPT group. This suggests that SPT already provides an excellent item-specific encoding that can hardly be improved on (Seiler and Engelkamp, 2003; Mulligan and Peterson, 2015; Kubik et al., 2014a,b).

For the *know* response, the results of Experiments 1 and 2 were not consistent. Experiment 1 showed that VT is more likely to engender *know* responses than SPT, but no such difference was evident in Experiment 2. It may be that because of the within-subjects design used in Experiment 1, the subjects tended to compare the items they learned in VT with those in SPT at the time of judgment. If the details of learning were less obvious in VT than in SPT, people might have tended to make *know* responses. However, there is no such situation in the between-subjects design.

## General discussion

In this study, we combined an SPT paradigm with a *remember/know* judgment paradigm and conducted two experiments to investigate the relations between item-specific processing and retrieval awareness. Experiment 1 used a *remember/know* paradigm to verify that SPT was accompanied by more re-collective retrieval. Experiment 2 examined whether item-specific processing improved the proportion of subsequent *remember* responses.

The results verified the hypothesis. Experiment 1 showed a stable SPT effect and that SPT engenders more *remember* responses, which means that SPT can increase the frequency of recalling re-collective experience. Experiment 2 confirmed these results and revealed that the item-specific processing could improve the recognition performance and increase the incidence of *remember* responses in the VT condition, but could not improve the memory performance or the incidence of *remember* responses under the SPT condition, which means that SPT can enhance item-specific processing.

Irrespective of whether a within-subjects or between-subjects design was used, both experiments obtained the result that SPT engenders more *remember* responses in the judgment, which is consistent with previous results (Conway and Dewhurst, 1995; Lövdén et al., 2002; Manzi and Nigro, 2008); that is, the SPT group more easily recalled re-collective experiences than the VT group. We conclude that the state of retrieval awareness is closely related to the attentional state in the encoding process. Previous studies have shown that people are more involved in events when performing actions (Kormi-Nouri, 2001). This is mainly reflected in the following two aspects: First, SPT requires participants to perform the action represented by the phrase, so they associated the phrase with themselves. Second, to perform actions better, people will pay attention only to action-related information and ignore unrelated environmental data (Engelkamp, 1995). This focused attention gives people more specific information and makes the memory traces more distinct, thereby leading people to remember performed actions more easily (Schatz et al., 2011).

However, the present result is *not* consistent with Gardiner et al's (2006) research showing that recognition performance and *remember* responses were not enhanced by SPT. One difference between our research and Gardiner's is that we used normal subjects, as opposed to developmental amnesiac subjects whose episodic memory was impaired and who failed to carry out the actions as effectively as the participants in the control group did.

The results of Experiment 2 supported the view that the item-specific processing leads to the retrieval awareness of SPT. First, previous studies have suggested that people's attention focuses on the verb, the noun, and the integration of the two in an action phrase to make the phrase distinct from other items (Steffens et al., 2006). The more specific information is encoded, the richer the clues that are retrieved. Thus, SPT can increase the frequency of recalling re-collective experience.

Second, VT can equally use item-specific and relational information, which leads to bad performance. However, when the VT group is given pleasantness-rating instruction, it breaks the balance of the use of item-specific and relational information maintained by VT under standard instruction, making VT use too much item-specific information and hindering the use of relational information. Previous studies have suggested that the item-specific processing can provide optimal encoding, thereby letting people achieve high memory performance without additional encoding strategies (Schatz et al., 2011) such as the conceptual strategy (Zimmer and Engelkamp, 1999). We have found that the pleasantness-rating task enhances the recognition performance in VT but not in SPT, indicating that SPT relies mainly on item-specific information.

Third, the incidence of *remember* responses was affected by the item-specific processing instruction. SPT exhibits a higher incidence of *remember* responses under standard instruction, whereas under pleasantness-rating instruction, VT and SPT show a similar incidence, which suggests that SPT contains item-specific processing already, and additional item-specific processing does not improve the incidence of *remember* responses.

Performing actions can enhance the processing of item-specific information. First, for a simple noun-verb phrase, subjects need to understand the meaning of the phrase and then physically enact it. Previous studies have suggested that producing words from definitions can lead to elaborative processing (MacLeod and Daniels, 2000), and generating actions from phrases is similar to this process. Second, the enactment can activate the corresponding mirror neurons (Glover and Dixon, 2013; Goldin-Meadow and Alibali, 2013) and form a motor imagery, which makes the action phrase more specific and distinct (Madan and Singhal, 2012b). Finally, the performing actions accompanied by a higher self-involvement level makes people focus their attention on action-relevant information rather than contextual information (Engelkamp, 1995; Kormi-Nouri, 1995), leading to more distinct memory traces. Hence, people can re-experience the original performed actions more frequently.

The present study does not support the result that SPT depends on relational information processing. If SPT depended on relational information processing, the item-specific processing would be missing and additional processing would be able to improve the performance of SPT like that of VT. However, this is not the case; performance in both recognition and *remember/know* judgment was unaffected by the additional item-specific processing. This is because, when the action is performed, the individual needs to focus on the action, and the situation cannot be similar to that of VT, where they can examine the associations among the phrases. Therefore, this research supports the view that SPT does not enhance or even hinder relational information processing (Engelkamp, 1995; Steffens, 2007).

## Conclusions

The conclusions of this study are as follows:

1. The SPT effect was obtained under standard instruction, but not under the pleasantness-rating instruction; that is, the pleasantness-rating instruction only improved the recognition performance for VT, suggesting that SPT provides an excellent item-specific encoding that can hardly be enhanced.
2. SPT allows *remember* responses more easily in *remember/know* judgment; that is, SPT can enhance the frequency of recalling re-collective experience. The pleasantness rating instruction significantly increased the incidence of *remember* responses for the VT group but not the SPT group, thereby suggesting that the greater number of *remember* responses in SPT was caused by item-specific processing.

## Ethics statement

This study was carried out in accordance with the recommendations of jilin university academic ethics committee with written informed consent from all subjects. All subjects gave written informed consent in accordance with the ethics committee.

## Author contributions

LW and GL designed research and wrote the paper. GL collected and analyzed the data.

### Conflict of interest statement

The authors declare that the research was conducted in the absence of any commercial or financial relationships that could be construed as a potential conflict of interest. The reviewer DB and handling editor declared their shared affiliation at time of review.

## Appendix

**Table A1:** The phrases used in the study.

**Learning phrases**		**Disturbing phrases**	
素描	Do a sketch	打开课本	Open a textbook
查字典	Consult a dictionary	拿起笔	Pick up a pen
擦黑板	Wipe a blackboard	做曲线	Make a curve
画三角形	Draw a triangle	记笔记	Take a note
削铅笔	Sharpen a pencil	交作业	Hand in homework
写字	Write a character	看报纸	Read newspaper
切白菜	Cut a cabbage	淘米	Wash rice
拧开煤气	Turn on the gas	洗芹菜	Wash celery
炒鸡蛋	Scrambled eggs	拍黄瓜	Smash cucumbers
包饺子	Make a dumpling	关电磁炉	Close an induction cooker
舀水	Scoop water	盛菜	Fill a dish
捣蒜	Beat garlics	加调料	Put condiment
拖地	Mop a floor	洒水	Spray water
扫纸屑	Sweep papers	倒垃圾	Take out trash
把杯口朝下	Turn the cup down	剪花枝	Cut flowers
捡起电池	Pick up a battery	挤干拖把	Squeeze a mop
开窗户	Open a window	拉吸尘器	Push vacuum cleaner
转动钥匙	Turn the key	锁自行车	Lock a bike
转方向盘	Turn a steering wheel	打开气囊	Open airbag
换档	Shift a gear	拧油盖	Screw a cap
拉起手闸	Pull up a hand brake	拉下窗帘	Pull down a curtain
擦车座	Clean a car	开车门	Open a car door
系安全带	Fasten seat belts	踩油门	Press an accelerator
撕开包裹	Tore a parcel	拉开钱包	Open a wallet
填收件人	Fill in a recipient	写信	Write a letter
查邮编	Zip a code	拆信封	Unpack an envelope
抹胶水	Spread glue	密封信件	Seal a letter
贴邮票	Stick a stamp	盖邮戳	Put seal on a stamp
付邮费	Pay postage	称重量	Check a weight
洗毛衣	Wash sweater	切蛋糕	Cut a cake
撒洗衣粉	Sprinkle washing powder	搅拌咖啡	Stir coffee
搓衣领	Rub a collar	倒开水	Pour water
拧毛巾	Wring a towel	削苹果	Peel an apple
擦盘子	Mop up a plate	端碗	Side bowl
刷鞋垫	Brush insoles	吃面条	Eat noodles
戴帽子	Wear a hat	拧开唇膏	Unscrew a lipstick
扣扣子	Button a coat	叠被子	Fold up a quilt
照镜子	Look in a mirror	穿袜子	Wear socks
挤牙膏	Squeeze toothpaste	擦皮鞋	Polish a shoe
抹桌子	Wipe a table	夹文件	Clip a file
刷牙	Brush teeth	系鞋带	Tie shoes
戴项链	Wear a necklace	拉拉链	Zip out coat
夹菜	Pick up vegetables	捞衣服	Fish out clothes
吸豆浆	Drink soya-bean milk	倒洗涤液	Pour liquid detergent
摇晃果汁	Shake juice	洗碗	Wash dishes
剥橘子	Peel an orange	打香皂	Apply a soap
掰馒头	Tear into steamed buns	涮筷子	Wash chopsticks
喝啤酒	drink beer	揉袖口	knead a cuff
